# PARP‐1 Inhibition Increases Oxidative Stress in Ets‐1‐Expressing MDA‐MB‐231 Breast Cancer Cells

**DOI:** 10.1002/cnr2.70119

**Published:** 2025-01-07

**Authors:** Magalie Hervieu, Arnaud J. Legrand, Emilie Floquet, Thierry Idziorek, Corentin Spriet, Didier Monté, Vincent Villeret, Marc Aumercier, Souhaila Choul‐li

**Affiliations:** ^1^ CNRS EMR9002 Integrative Structural Biology Lille France; ^2^ Univ. Lille, Inserm, CHU Lille, Institut Pasteur de Lille U1167‐RID‐AGE‐Risk Factors and Molecular Determinants of Aging‐Related Diseases Lille France; ^3^ Univ. Lille, CNRS, Inserm, CHU Lille UMR9020‐U1277‐CANTHER‐Cancer Heterogeneity Plasticity and Resistance to Therapies Lille France; ^4^ Univ. Lille, CNRS, Inserm, CHU Lille, Institut Pasteur de Lille, US41‐UMS2014‐PLBS, F‐59000, Lille, France; Univ. Lille, CNRS UMR8576‐UGSF‐Structural and Functional Glycobiology Unit Lille France; ^5^ Département de Biologie, Faculté des Sciences Université Chouaïb Doukkali El Jadida Morocco

**Keywords:** breast cancer cells, DNA damage, Ets‐1 transcription factor, oxidative stress, PARP‐1 inhibitors

## Abstract

**Background:**

The Ets‐1 transcription factor plays a primordial role in regulating the expression of numerous genes implicated in cancer progression. In a previous study, we revealed that poly(ADP‐ribose) polymerase‐1 (PARP‐1) inhibition by PJ‐34 results in Ets‐1 level increase in cells, which is related with cell death of Ets‐1‐expressing cancer cells.

**Aims:**

The mechanism of the antitumor effect of PARP‐1 inhibition was investigated in the Ets‐1‐expressing MDA‐MB‐231 breast cancer cells.

**Methods and Results:**

We tested the effects of four PARP inhibitors (PARPi) (PJ‐34, Veliparib, Olaparib, and Rucaparib). We first demonstrated that PARPi reduced cells growth through G2/M cell cycle arrest. Next, we evaluated PARP‐1 inhibition effect on oxidative DNA damage in Ets‐1‐overexpressing and Ets‐1‐non‐expressing breast cancer cells and we showed that PARPi led only Ets‐1‐overexpressing cells to accumulate it, which triggers the DNA damage response as revealed by the increase in the level of a panel of DNA damage‐related proteins. Importantly, we demonstrated that PARPi increased reactive oxygen species (ROS), only in Ets‐1‐overexpressing cells and this is accompanied by upregulation of p47^phox^ expression, a subunit of the NAPDH oxidase (NOX).

**Conclusion:**

These preliminary findings correlate PARPi‐induced oxidative DNA damage/oxidative stress to Ets‐1 expression in breast cancer cells.

## 1 Introduction

The PARP‐1 protein is a nuclear enzyme implicated in cellular responses to oxidative stress, mainly through DNA damage repair. Indeed, oxidative stress induces DNA damage, which in turn activates PARP‐1. Once activated, PARP‐1 adds ADP‐ribose polymers to the target proteins, thereby stabilizing DNA repair complexes at the damaged DNA site [1]. In this way, PARP‐1 repairs diverse single‐strand and double‐strand DNA damages like in homologous recombination (HR), nonhomologous end joining (NHEJ), and nucleotide excision repair (NER) [2]. Due to its role in DNA repair, PARP‐1 expression is increased in several types of cancers (e.g., breast cancers) [3] that have high levels of DNA damage, promoting thus tumor progression [4]. To prevent DNA repair process in cancer cells, numerous PARPi have been developed and initially used in combination with chemo‐ and radiotherapy in clinical trials [5, 6]. In the following strategy, PARPi are used as a monotherapy against cancer cells with a specific gene alteration [7]. In fact, during DNA replication, double‐strand breaks (DSBs) are repaired by HR, which requires BRCA. Cells that are deficient in BRCA require PARP‐1 for the repair of DSBs. Thus, the inhibition of PARP‐1 in BRCA1/2‐deficient cells causes cell death through intense DSBs formation [8]. This “synthetic lethality” effect of PARP‐1 inhibition is effective in breast cancer with BRCA mutations, but not in breast cancer with wild‐type BRCA [9, 10] and is enlarged to cover cancer cells showing other HR protein deficiencies [11]. DNA repair proteins deficiency is not always a marker of PARPi sensitivity; an increase in certain proteins level can also be a marker, as in the case of the ETS proteins.

Ets‐1 oncoprotein is the first characterized member of the ETS family [12]. It activates the transcription of numerous genes implicated in various biological processes, including tumor progression [13]. In a number of breast cancers, Ets‐1 is overexpressed [14], which is correlated with a poor prognosis [15, 16]. Indeed, Ets‐1 has been demonstrated to play an important role in the progression of breast cancer by promoting metastasis/invasion, epithelial‐to‐mesenchymal transition (EMT), and neo‐angiogenesis [17]. The involvement of Ets‐1 in the invasiveness and metastasis of breast cancer cells is associated with the transcriptional activation of genes encoding matrix metalloproteinases (MMPs), proteins that degrade the extracellular matrix, such as MMP1, MMP3 and MMP9 [18, 19, 20], and plasminogen activator inhibitor‐1 (PAI‐1), an inhibitor of urokinase‐type plasminogen activator (uPA) that promotes cells migration [14, 21]. The promotion of metastasis by Ets‐1 is mediated by the YAP (Yes‐associated protein) signaling [16]. With regard to the role of Ets‐1 in the promotion of EMT, this is mediated by the induction of ZEB1/2 expression, which are transcription factors characterizing EMT [22, 23].

Previous studies have identified PARP‐1 as a physical interaction protein of the ETS fusion factors, Fli1 and Erg, and have considered these ETS fusion proteins as predictors of sensitivity to PARPi in many cancers [24, 25, 26, 27]. Indeed, PARP‐1 inhibition potentiates DNA damage caused by the overexpression of EWS/Fli‐1 and TMPRSS2/Erg chimeric proteins in Ewing's sarcoma and prostate cancer, respectively. This leads to high inhibition of cancer progression [24, 25, 26, 27]. Likewise, our previous studies have demonstrated that PARP‐1 interacts physically with Ets‐1 and negatively regulates its level in cancer cells via PARylation [28]. In addition, PARP‐1 inhibition led to an increase of the transactivation activity of Ets‐1. This induced cell death of Ets‐1‐expressing cancer cells and an increase in DNA damage as detected by the level of γH2AX, a biomarker for DSBs [28].

The present study reports the possible mechanism through which PARP‐1 inhibition induces death of MDA‐MB‐231 breast cancer cells line. We first evaluated the effect of PARP‐1 inhibition in cell growth and cell cycle progression. Next, we analyzed the accumulation of oxidative DNA lesions and the levels of DNA damage‐related proteins in Ets‐1‐overexpressing and Ets‐1‐non‐expressing breast cancer cells after treatment with different PARPi. The effect of PARPi in ROS production is then evaluated. This preliminary study supports the effectiveness of PARPi in Ets‐1‐expressing breast cancer cells and reveals the possible mechanism of oxidative DNA damage induction through PARP‐1 inhibition.

## 2 Materials and Methods

### 2.1 Cell Culture/PARP‐1 Inhibition

MDA‐MB‐231 and MCF‐7 cells (CLS, Eppelheim, Germany) were cultured in Dulbecco's modified Eagle's medium (DMEM; Invitrogen, Life Technologies, Saint Aubin, France) supplemented with 10% fetal bovine serum (FBS; Invitrogen) and 50 μg/mL gentamycin (Invitrogen) at 37°C in a humidified 5% CO_2_ incubator. To inhibit PARP‐1 activity, cells were incubated in medium supplemented with PARP‐1 inhibitor for 48 h. As positive control for oxidative stress‐induced ROS production, the cells were incubated for 48 h with 200 μM hydrogen peroxide (H_2_O_2_).

### 2.2 Pharmacological Inhibition of PARP‐1

We used four different PARPi. PJ‐34 (N‐(6‐Oxo‐5,6‐dihydrophenanthridin‐2‐yl)(N,N‐dimethylamino) acetamide hydrochloride) and ABT‐888 ((R)‐2‐(2‐methylpyrrolidin‐2‐yl)‐1H‐benzo[d]imidazole‐4‐carboxamide; veliparib) were purchased from Enzo Life Sciences (Villeurbanne, France) and were used at 20 μM and 2 μM concentration, respectively. Olaparib (AZD‐2281 or KU 58948) and rucaparib (AG‐014699) were purchased from Selleckchem (Houston, TX, USA) and were used at 10 μM concentration based on the literature data [29, 30].

### 2.3 MTT Assay

Cells were seeded into 96‐well‐plate (3300 cells per well) and cultured overnight before the experiment. After 48 h of PARPi treatment, the cell culture medium was removed and MTT solution (3‐(4,5‐Dimethylthiazol‐2‐yl)‐2,5‐Diphenyltetrazolium Bromide; Life Technologies) were added at 1 mg/mL in phosphate‐buffered saline (PBS). After 3 h incubation, MTT solution was eliminated and replaced by dimethyl sulfoxyde (DMSO) to solubilize formazan crystals and absorbance was measured by optical density at 570 nm. Results are expressed as a percentage of the control values.

### 2.4 Flow Cytometric Analysis

Cells seeded into 24‐well plates (19 000 cells/well) were incubated overnight in medium containing 10% FBS, and then treated with PARPi. After trypsinization and PBS wash, we fixed cell with cold 70% ethanol. Next, we stained cells with propidium iodide (PI) (1 mg/mL) after RNase A treatment (100 μg/mL). Cell cycle phase distributions were determined on a BD LSRFortessaTM X‐20 cell analyser (BD Biosciences) and examined using Kaluza analysis software (Beckman Coulter).

### 2.5 Cell Lysates

After washing twice with cold PBS 1X solution, cells were resuspended in 200 μL of RIPA lysis buffer (Sigma‐Aldrich) with protease inhibitors cocktail. The solutions were then briefly vortexed and incubated on ice for 30 min. Lysates were clarified by centrifugation at 20000 g, for 15 min at 4°C. Before Western blot analysis, yields were measured by colorimetry (Bio‐Rad assay).

### 2.6 Western Blot

We performed Western blot using 30 μg of total cell lysate proteins, which were boiled in Laemmli buffer (50 mM Tris, pH 6.8, 2% SDS, 5% 2‐mercaptoethanol, 10% glycerol, 0.1% Bromophenol Blue) and resolved by SDS/PAGE. Proteins in gels were transferred on to a HybondTM‐C Extra membrane (Amersham Biosciences) and blocked for 1 h at room temperature (25°C) in 5% (w/v) non‐fat dried skimmed milk powder in PBS. The membrane was then incubated with the primary antibody for 1 h at room temperature in blocking buffer. Primary antibodies utilized were mouse C‐4 anti‐β‐Actin, rabbit C‐20 anti‐Ets‐1, H‐300 anti53BP‐1 and anti‐Rad51 (Santa Cruz Biotechnology, Germany), anti‐8‐oxoguanine (Millipore, USA), anti‐p47^phox^ (Cell Signaling, USA), anti‐γH2AX (phospho S139) (Abcam, France), and anti‐PAR from Enzo Life Sciences (Villeurbanne, France). The washed membrane was then incubated for 1 h at room temperature with HRP (horseradish peroxidase)‐conjugated secondary antibody (Santa Cruz Biotechnology) in blocking buffer. Bound antibodies were visualized using the Western LightningTM chemiluminescence detection system (PerkinElmer Life Sciences Biotechnology). Reprobing was performed using the Re‐Blot Plus Strong Antibody Stripping Solution (Millipore) according to the manufacturer's instructions.

### 2.7 Immunofluorescence

MDA‐MB‐231 cells (9000, cells /well), treated with PARPi, were grown on sterile 17 mm glass coverslips precoated with 40 μg mL‐1 poly‐L‐lysine (Sigma) until they reached 90% confluence. Cells were washed, fixed with 4% paraformaldehyde for 15 min, and permeabilized with 0.1% Triton X‐100 for 4 min at room temperature. Cells were then incubated for 30 min at room temperature with mouse C‐4 anti‐Ets‐1 (Santa Cruz Biotechnology), rabbit anti‐γH2AX (phospho S139), mouse anti‐8‐oxoguanine and rabbit anti‐Rad51 diluted 1:500 in PBS (pH 7.5) containing 1% bovine serum albumin (BSA). After washings, antibody binding was detected with goat anti‐mouse Alexa Fluor 488 or goat anti‐rabbit Alexa Fluor 594 (Invitrogen) at a 1:500 dilution for 30 min at room temperature in the dark. DAPI (4,6‐diamidino‐2‐phenylindole; Santa Cruz Biotechnology, 2 μg/mL) was utilized to counterstain nuclei in UltraCruz Mounting Medium for 1 min at room temperature. Fluorescent signals were analyzed with a confocal laser‐scanning microscope (Nikon A1‐R, Nikon Instruments). γH2AX and Rad51 foci in the nuclei were counted by evaluating local maxima in fluorescence intensity. A minimum of 100 nuclei were examined, and those with 10 foci or more were considered to be positive.

### 2.8 ROS Detection in Cells

The fluorescent dye dihydroethidium (DHE; Life Technologies) was utilized to evaluate superoxide production in cultured cells. Briefly, cells (9000 cells/well) were cultured onto glass chamber slide (Nunc) and treated with PARPi. After 24 h, cells were incubated with 10 μM DHE for 15 min at 37°C, washed with 0.1 M phosphate buffer (pH 7.4) and fixed with 4% paraformaldehyde for 15 min at room temperature. A confocal laser‐scanning microscope (Nikon A1R, Nikon Instruments) was used to observe fluorescence.

### 2.9 RNA Extraction/Reverse Transcription‐Quantitative Polymerase Chain Reaction (RT‐qPCR) Assay

RNA extraction was performed using TRIzol Reagent (Invitrogen). OligodT(Eurogentec) and reverse transcriptase (MMLV, Invitrogen) were used to reverse transcribe 1 μg of RNA into cDNA. qPCRs were carried out on Stratagene Mx3000 using Fast PlusEvaGreen qPCR Master mix‐low ROX (Interchim, Montluçon, France) with 500 ng of cDNA and with 125 ng of forward and reverse primers for each of two genes: *p47* ^*phox*^, 5’‐ACAGTCCTGACGAGACGGAA‐3′ and 5’‐GTGACGTCGTCTTTCCTGATG‐3′; and glyceraldehyde‐3‐phosphate dehydrogenase (*gapdh*), 5’‐GGAGCGAGATCCCTCCAAAAT‐3′ and 5’‐GGCTGTTGTCATACTTCTCATGG‐3′. All PCRs were run for 35 cycles: 94°C/30 s for DNA denaturation, 55°C/30 s for primers hybridization, and 72°C/30 s for elongation. PCRs were normalized to *gapdh* and run in triplicate for each sample. Delta/delta threshold cycle method (2‐^ΔΔCT^) was utilized to calculate fold changes in mRNA levels and each group was normalized to the mRNA level of untreated cells.

### 2.10 Statistical Analysis

GraphPad Prism 6.0 (GraphPad Software Inc., USA) was used for all treatment of data. To evaluate significant differences between two groups (untreated and PARPi‐treated cells), Student's t test (two‐tailed) was utilized. A P‐value of 0.05 or below is used to define statistical significance. *P*‐value of 0.05, 0.01, 0.001, and 0.0001 are mentioned by *, **, ***, and ****, respectively. Each experiment was reproduced three times. Values were reported as mean ± SD or box plots.

## 3 Results

### 3.1 PARP‐1 Inhibition Leads to Reduced Proliferation of MDA‐MB‐231 Cells Through G2/M Cell Cycle Arrest

**FIGURE 1:**
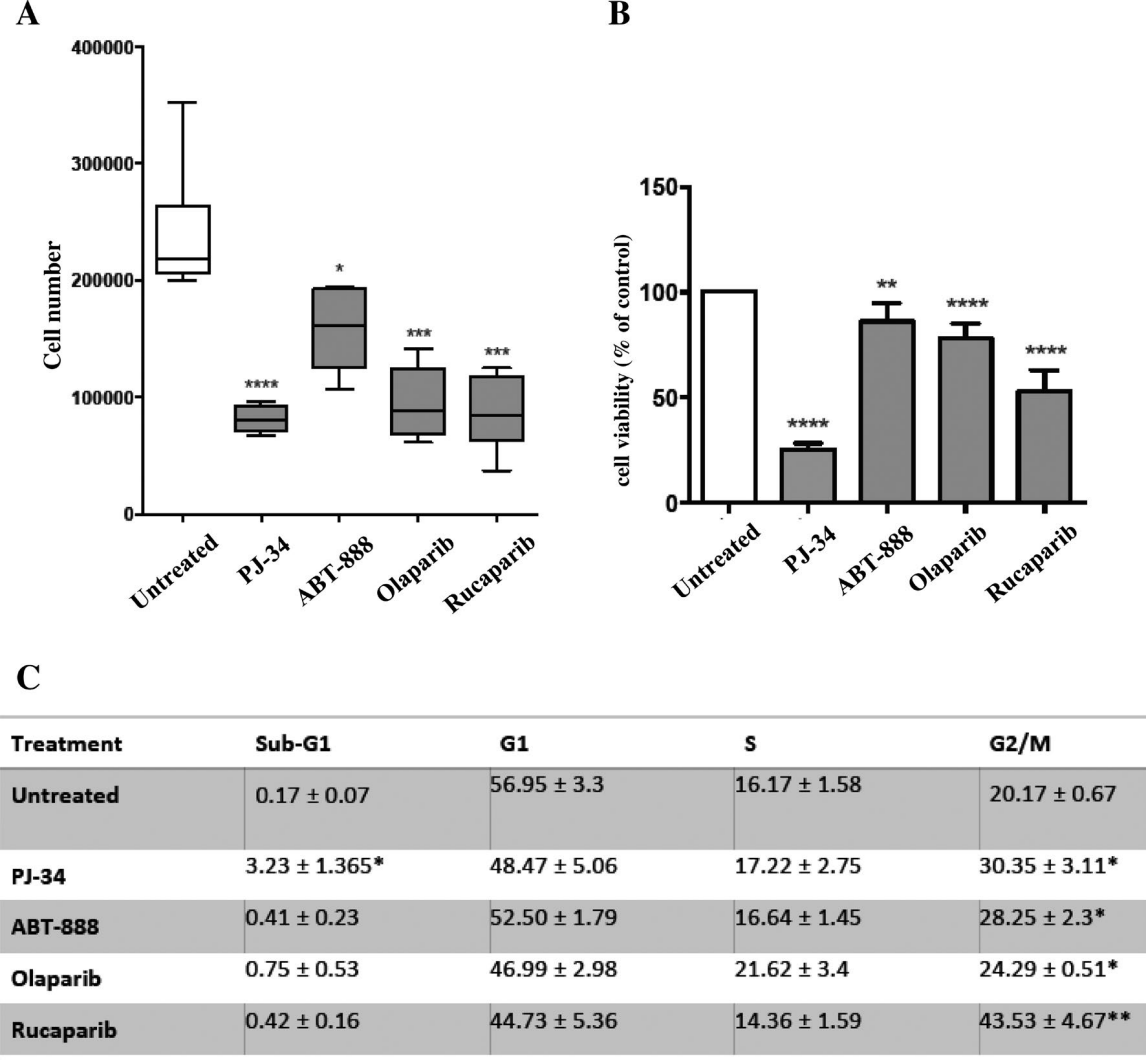
PARP‐1 inhibition decreases MDA‐MB‐231 cells proliferation through G2/M arrest. (A) Proliferation activity by cell counting in cells treated with PARPi (PJ‐34 (20 μM), ABT‐888 (2 μM), olaparib (10 μM) or rucaparib (10 μM)) for 48 h. (B) MTT assay in cells treated with PARPi. Viable cell counts are expressed as percent values of untreated cells. **p < 0.01 and ****p < 0.0001. (A–B) Results are mean of six experiments performed in triplicate. (C) Cell cycle distribution analysis of cells treated with PARPi by Flow cytometry using PI as probe. Percentages of fluorescent cells in Sub‐G1, G1, S, and G2/M were reported. Results are the mean ± S.D. of three separate experiments. *p < 0.05 and **p < 0.01.

Our previous work demonstrated that PARP‐1 inhibition by PJ‐34 induced cell death of Ets‐1‐expressing cancer cells [28]. Here, we decided to extend this previous research by evaluating the effects of PARPi on the growth and cell cycle progression of breast cancer cells using three other well‐studied PARPi: olaparib and rucaparib, approved by FDA for the treatment of advances breast cancers with mutated‐BRCA1/2 [31], and ABT‐888 (veliparib), currently under evaluation in clinical trials [32]. To do so, the cell count and MTT assay were performed using the aggressive MDA‐MB‐231 breast cancer cell line, which endogenously overexpress Ets‐1 and is highly invasive and metastatic [33, 34]. Cell count results demonstrate that treatment of MDA‐MB‐231 cells with PARPi was correlated with a reduction of cell number compared to untreated control (Figure 1A). PJ‐34 and rucaparib were more effective in decreasing cell number than olaparib and ABT‐888. MTT assay analysis demonstrated a reduction of cell viability with PARPi treatment (Figure 1B). PJ‐34 was highly effective in decreasing cellular viability compared with the control group. Rucaparib was the second most active agent. Finally, olaparib and ABT‐888 were the least effective agents.

To understand the mechanism behind the anti‐proliferative effect of PARPi, a cell cycle analysis was performed to examine how PARPi treatment influences cell cycle progression (Figure 1C). The result showed that rucaparib, PJ‐34 and to lesser extent ABT‐888 and olaparib induced increases in the G2/M population in comparison to non‐treated cells (i.e., 20.17%). Moreover, the percentage of PJ34‐treated cells in the sub‐G1 phase (apoptotic cells) was enhanced significantly compared to control cells. Thus, our data showed that the inhibition of PARP‐1 reduces MDA‐MB‐231 proliferation and induces the arrest of cell cycle at G2/M transition.

### 3.2 PARP‐1 Inhibition Increases Oxidative DNA Damage in Breast Cancer Cells Expressing Ets‐1

**FIGURE 2:**
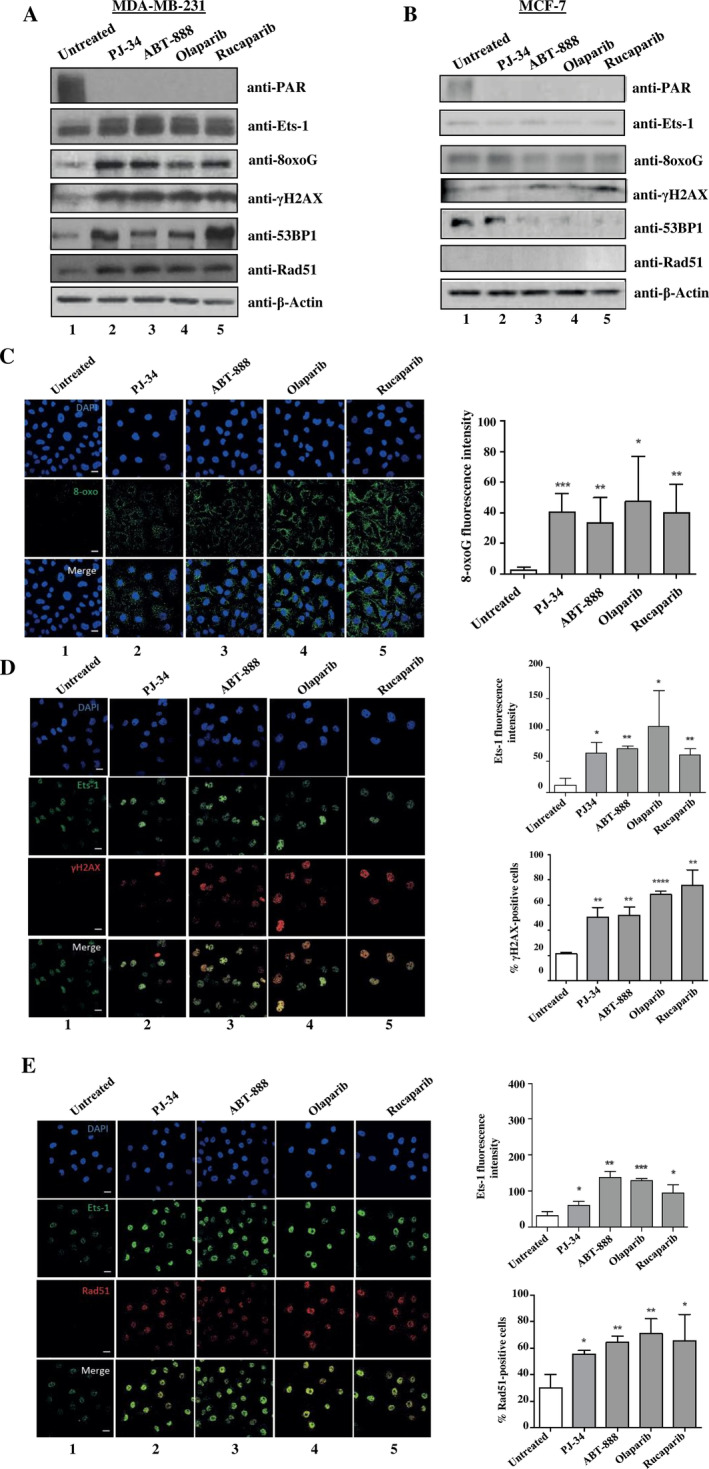
PARP‐1 inhibition leads to an increase of oxidative DNA damage in cells expressing Ets‐1. (A, B) The effect of PARP‐1 inhibition on oxidative DNA lesions and DNA damage response were examined by Western blot analysis using antibodies anti‐PAR (a product of PARP‐1 activation), anti‐Ets‐1, anti‐8oxoG, anti‐γH2AX, anti‐53BP1, anti‐Rad51, and anti‐β‐Actin. (C–E) Immunofluorescence of 8‐oxoG (C), γH2AX (D), and Rad51 (E) in MDA‐MB‐231 treated by PARPi. 8‐oxoG is visualized in green (C), γH2AX and Ets‐1 are visualized in red and green, respectively (D), and Ets‐1 is visualized in green and Rad51 is visualized in red (E). γH2AX‐positive and Rad51‐positive cells correspond to cells with > 10 γH2AX foci and > 10 Rad51 foci, respectively. 8‐oxoG fluorescence intensity (C) and the percentage ofγH2AX‐positive (D) and Rad‐51‐positive cells (E) are shown in the accompanying graphs. More than 100 cells from three separate immunofluorescence experiments are counted and evaluated to carry out statistical analysis. DAPI staining was used to show nuclei. Cells were evaluated with confocal microscopy at ×40 magnification. *p < 0.05, **p < 0.01, ***p < 0.001, and ****p < 0.0001. Scale bar = 20 μm.

To test whether PARP‐1 inhibition induces oxidative DNA damage, we assessed the level of 7,8‐dihydro‐8‐oxoguanine (8‐oxoguanine or 8‐oxoG), a marker for oxidative base damage by Western blot analysis in Ets‐1‐overexpressing MDA‐MB‐231 cells. PARP‐1 inhibition with PJ‐34, olaparib, ABT‐888, or rucaparib was associated with an increase in the level of Ets‐1 protein and the accumulation of 8‐oxoG lesions (Figure 2A, lanes 2–5) compared to untreated control (Figure 2A, lane 1). Additionally, we examined whether the PARP‐1 inhibition activated the DNA damage response by evaluating the biomarkers for DNA DSBs: phosphorylated H2AX (γH2AX), p53‐binding protein 1 (53BP1), and Rad51. PARPi led to a greater γH2AX signal (Figure 2A, lanes 2–5), as we previously demonstrated with PJ‐34 [28], and also to higher levels of 53BP1 and Rad51 expression (Figure 2A, lanes 2–5). We next performed the same experiment using MCF‐7, a human breast cancer cells, which have been proved to express a very low levels of Ets‐1 [35, 36]. Addition of PARPi to MCF‐7 cells does not affect the levels of 8‐oxoG, γH2AX, 53BP1 nor Rad51 in Western blot (Figure 2B). We may suggest that PARP‐1 inhibition increased oxidative DNA damage only in breast cancer cells expressing Ets‐1.

To confirm Western blot results, we carried out immunofluorescence experiments to analyze the accumulation of 8‐oxoG lesions and γH2AX/Rad51 expression after PARPi treatment in MDA‐MB‐231 cells. Results revealed that PARPi‐treated cells exhibited higher 8‐oxoG levels compared to untreated cells (Figure 2C, left panel, lanes 2–5). Statistical analysis shown significant increases in 8‐oxoG fluorescence intensity: 18‐fold (*p* < 0.05) after olaparib treatment; and 13‐fold and 15‐fold after ABT‐888 and rucaparib treatment (**p < 0.01), respectively. Moreover, 8‐oxoG formation was significantly higher with PJ‐34 treatment compared to untreated cells (***p < 0.001) (Figure 2C, graph). For DSBs‐related proteins expression, PARPi highly increased the number of γH2AX foci (Figure 2D, left panel, lanes 2–5). Statistical analysis showed that γH2AX‐positive cells increased significantly: 2.4‐fold with PJ‐34 and ABT‐888 (** p < 0.01), 3.2‐fold with olaparib (****p < 0.0001) and 3.6‐fold with rucaparib (**p < 0.01) (Figure 2D, graph). Immunofluorescence for Rad51 revealed an increase in the number of cells that were positive for Rad51 after PARPi treatment (Figure 2E, left panel, lanes 2–5). Statistical analysis of this experiment revealed a significant 1.8‐fold and 2.2‐fold increase in Rad51‐positive cells with PJ‐34 and rucaparib, respectively (*p < 0.05), 2.1‐fold with ABT‐888 and 2.4‐fold with olaparib (**p < 0.01) (Figure 2E, graph). A high increase of the level of Ets‐1 in cells after PARPi treatment was showed (Figure 2D,E) and this was confirmed by statistical analysis. We suggest that the inhibition of PARP‐1, which it is known to induce Ets‐1 accumulation [28], increases oxidative DNA damage only in breast cancer cells expressing Ets‐1.

### 3.3 PARP‐1 Inhibition Increases ROS Production Only in Ets‐1‐Expressing Cells, Which Is Accompanied by Upregulation of p47^phox^, a Subunit of the NOX Complex

**FIGURE 3:**
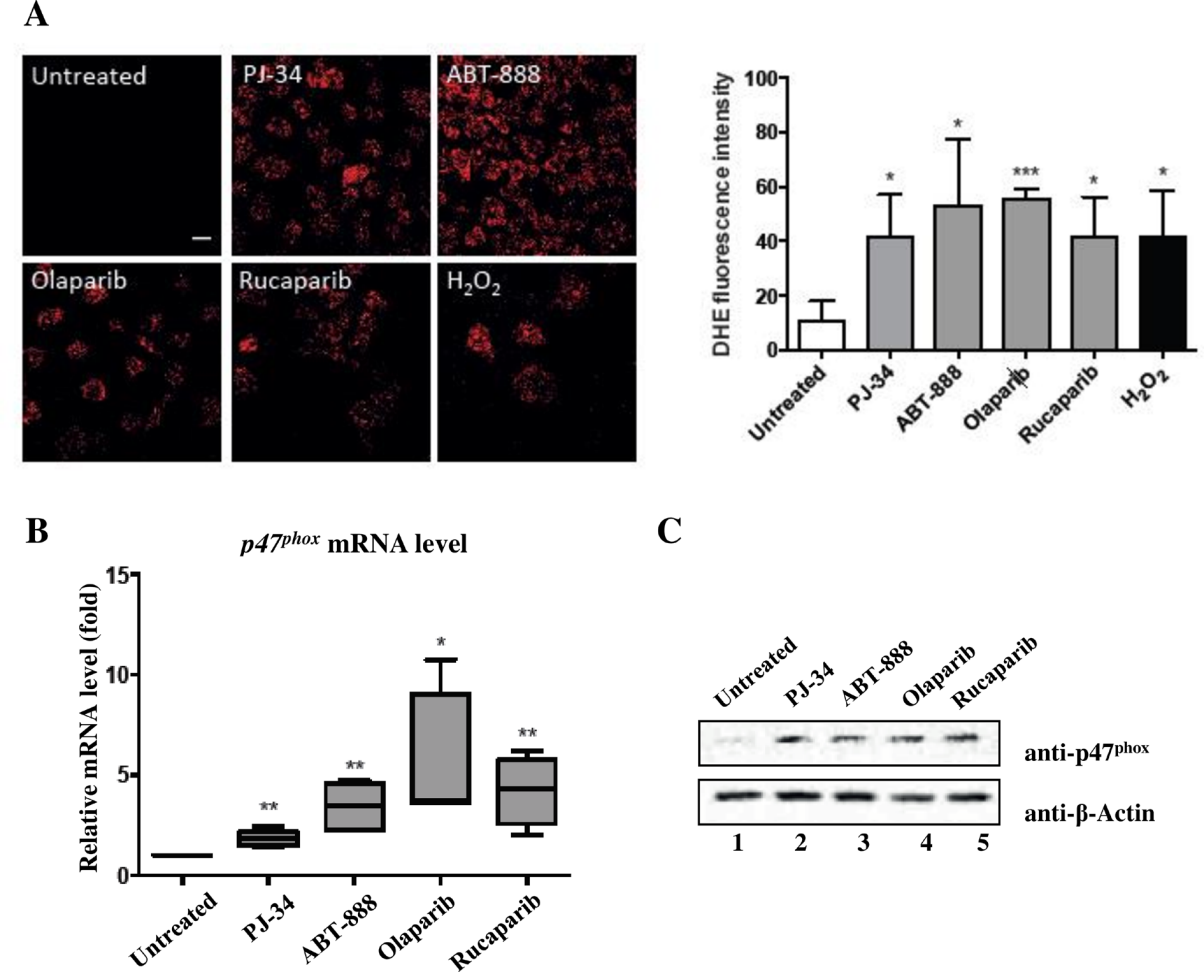
Inhibition of PARP‐1 increased ROS production in Ets‐1‐expressing cells, which is accompanied by an enhancement of NOX activity. (A) Visualization of ROS with a confocal microscope (× 40) in MDA‐MB‐231 cells treated with PARPi (ABT‐888 (2 μM), PJ‐34 (20 μM), Olaparib (10 μM), Rucaparib (10 μM)). H_2_O_2_ treatment (200 μM) was used as positive control. Scale bar = 20 μm. The fluorescent signal was measured and quantified. The data reflect the mean ± S.D. of three separate experiments. *p < 0.05, **p < 0.01, and ***p < 0.001. (B) RT‐qPCR measuring *p47* ^*phox*^ mRNA levels in MDA‐MB‐231 treated with PARPi. Comparative threshold cycle 2‐^ΔΔCT^ method was used to normalize measurements to *gapdh* mRNA levels. Box plots represent four independent tests carried out in triplicate. *p < 0.05 and **p < 0.01. (C) Western blot of p47^phox^ protein in MDA‐MB‐23 cells treated with PARPi using anti‐p47^phox^ and anti‐β‐Actin antibodies. Representative blots are shown (*n* = 3).

To test whether ROS are involved in the pathway through which PARP‐1 inhibition induces oxidative DNA damage in Ets‐1‐overexpressing cells, ROS levels were examined in MDA‐MB‐231 and MCF‐7 cells by the fluorescent dye DHE staining. From the resulting images (Figure 3A, left panel) and from quantitation of DHE fluorescence intensity (Figure 3A, right panel), it can be inferred that intracellular levels of ROS were increased in MDA‐MB‐231 cells treated with the four PARPi. In MCF‐7 cells, addition of PARPi did not have any effect on ROS level in DHE staining (data not shown).

Turning next to a possible mechanism by which PARP‐1 inhibition may increase ROS in MDA‐MB‐231. Since PARP‐1 inhibition increases ROS production only in cells expressing Ets‐1, we can speculate that this may be mediated by Ets‐1‐driven transcriptional activity. Indeed, Ets‐1 is known to stimulate ROS generation by upregulating the expression of p47^phox^, a subunit of the NOX complex [37], a main producer of cellular ROS [38]. To test the effect of PARPi on p47^phox^ expression in MDA‐MB‐231, we performed RT‐qPCR analysis and we observed an induction of *p47* ^*phox*^ mRNA expression in treated cells with PJ‐34, ABT‐888 and rucaparib (**p < 0.01) and with olaparib (*p < 0.05), whereas no increase in *p47* ^*phox*^ mRNA expression was observed in untreated cells (Figure 3B). The upregulation of *p47* ^*phox*^ was confirmed at protein level (Figure 3C). These results suggest that upregulation of p47^phox^ might, in part, mediate the increase of ROS when PARP‐1 is inhibited in Ets‐1‐expressing MDA‐MB‐231 breast cancer cells.

## 4 Discussion

PARPi are of in increasing interest in breast cancer therapy. They are effective in breast cancer cells lacking BRCA1/2 and other HR repair genes [8, 39]. However, relatively limited research has been conducted to investigate the efficacy of PARPi therapy in BRCA1/2 wild‐type breast cancer. The approval of niraparib for the treatment of ovarian cancer without BRCA1/2 mutations suggests a potential efficacy of PARPi in other BRCA wild‐type cancers [40]. This may depend on identifying biomarkers that can predict the sensitivity of cancer cells to PARPi.

In this study, we examined the effect of four PARPi, PJ‐34, ABT‐888, olaparib, and rucaparib in two breast cancer cell lines without BRCA mutation, Ets‐1‐expressing MDA‐MB‐231, and Ets‐1‐non‐expressing MCF‐7. Given the implication of PARP‐1 in gene expression regulation, the transcriptome profile is used to evaluate the response to PARP inhibitors. Indeed, PARP‐1 regulates the transcriptional activity of many factors involved in cancer (e.g., Ets‐1) [41]. Thus, deregulation of transcription can sensitize cancer cells to PARPi, as we previously demonstrated for the Ets‐1 factor, which accumulates in cells and whose transcriptional activity is increased by the inhibition of PARP‐1 [28]. Here, we show that PARPi increase oxidative DNA damage in Ets‐1‐expressing MDA‐MB‐231 cells, but not in Ets‐1‐non‐expressing MCF‐7 cells. This is revealed by the evaluation of a marker for oxidative base damage, 8‐oxoG, and biomarkers for DNA DSBs, γH2AX, 53BP1, and Rad51. It is worth noting that although Cruz et al. have correlated Rad51 foci to PARPi resistance in BRCA‐mutated breast cancer [42], others have considered Rad51 upregulation as a marker for PARPi sensitivity beyond BRCA mutation [43, 44]. We suggest that the lack of PARP‐1 activity increases single‐strand breaks (SSBs) which become DSBs when DNA replicates. This result is in accordance with other studies carried out on ETS family members, showing that under PARP‐1 inhibition, strong expression of EWS/Fli1, and Erg induces a high increase in DSBs. The increase of DSBs is conditioned by ETS proteins expression, as depletion of ETS proteins suppresses DSBs after the inhibition of PARP‐1. These results have led the authors to suggest that ETS fusion factors may represent a biomarker to predict the cell response to PARPi [24, 25, 26].

The possible mechanism of increased oxidative DNA damage under PARP‐1 inhibition is invistigated in the present report. Indeed, we demonstrated that ROS levels are increased by PARPi only in Ets‐1‐overexpressing MDA‐MB‐231 cells. In addition, we found that p47^phox^ was up‐regulated by PARPi. It has been reported that Ets‐1 transcriptional activity is increased when the catalytic activity of PARP‐1 is inhibited [28], and Ets‐1 is known to stimulate the production of ROS by increasing the expression of NOX complex components such as p47^phox^ [45]. We can therefore speculate that the higher Ets‐1 transcriptional activity stimulates ROS generation by increasing the activity of the NOX complex, in part by increasing of p47^phox^ expression. This not exclude that other Ets‐1 target genes implicated in ROS production may mediate the effect of PARP‐1 inhibition. For example, Ets‐1 is known to up‐regulate the expression of Aldehyde oxydase 1 (Aox1) [46, 47], an enzyme that produces hydrogen peroxide and promotes ROS production [48]. We can therefore suggest that the enhanced Ets‐1 transcriptional activity mediated by PARP‐1 inhibition leads to excessive ROS production by upregulating Aox1 expression. It is also possible that altered expression of Ets‐1 target genes other than those directly involved in ROS generation may also contribute to the effect of PARP‐1 inhibition. Indeed, Ets‐1 transactivates the expression of matrix metalloproteinase3 (MMP3) [49], an enzyme known to increase NOX complex activity by activating the expression of Rac1, another member of NOX complex [50]. It will be interesting to investigate whether these Ets‐1‐regulated pathways can explain the effect of PARP‐1 inhibition.

It is known that cancer cells present elevated ROS level to ensure their survival. However, excessive ROS production may induce their death [51]. High ROS generation by the NOX complex produces oxidative DNA damage that induces serious effects for cell survival [45]. Thus, high levels of ROS may generate SSBs that stop replication forks during DNA replication, leading to DSBs that are repaired predominantly by the HR repair pathway via BRCA1/2. Interestingly, Ets‐1 has been shown to repress BRCA1/2 expression in MDA‐MB‐231 and its knockdown in these cells upregulates BRCA1/2 expression and limits the sensitivity to olaparib [52]. Therefore, DSBs are not repaired by the inhibited PARP‐1, nor by BRCA1 and BRCA2 wild‐types whose expression is repressed by Ets‐1. This may prevent the HR repair pathway, which can led to genomic instability and cell death [53].

In addition to breast cancer, PARP‐1 is overexpressed and the PARylation level is increased in various other cancers, such as ovarian [54] and colorectal [55] cancers, thereby promoting tumor progression. In ovarian cancer, where PARP‐1 expression is correlated with a poor prognostic, its inhibition has been shown to induce oxidative stress, which mediates its antitumor effect [54]. This effect is the consequence of the upregulation of NOX1 and NOX4, which results in an increase in ROS production. Given that Ets‐1 is known to be overexpressed in ovarian cancer and has been suggested as a poor prognostic factor [56, 57], it seems reasonable to speculate that the oxidative stress‐mediated antitumor effect, observed after PARP‐1 inhibition, is the result of the transcriptional activity of Ets‐1.

The effectiveness of PARPi in only Ets‐1 expressing breast cancer cells lead us to suggest that Ets‐1 expression may represent a biomarker potentially able to identify the most sensitive breast cancer cells for treatment with PARPi. Further investigation of this concept may facilitate the broader utilization of PARPi in other Ets‐1‐expressing carcinomas, and potentially even in Ets‐1‐expressing leukemias. The prospective therapeutic applications are indeed significant.

Some limitations of this study should be noted. The anti‐proliferative effect and the cell cycle arrest of PARPi may be due to the oxidative stress, as they may also be due to other mechanisms. Additional studies with antioxidants are required to evaluate whether they can rescue the anti‐proliferative effect of PARPi. Furthermore, we have shown a correlation between Ets‐1 expression and oxidative DNA damage/oxidative stress. Further studies investigating Ets‐1 expression as a direct cause are of major importance. In this sense, it is interesting to test whether Ets‐1 knockdown can cancel the PARPi effect in Ets‐1‐expressing breast cancer cells. Specific inhibition of p47^phox^ is also worth testing to confirm that PARPi‐induced oxidative DNA damage/oxidative stress is mediated by the upregulation of p47^phox^ expression.

## 5 Conclusion

We studied in vitro activity of four PARPi in Ets‐1‐expressing MDA‐MB‐231 and Ets‐1‐non‐expressing MCF‐7 breast cancer cell lines and we demonstrated that PARP‐1 inhibition increases oxidative DNA damage and oxidative stress only in MDA‐MB‐231. This is accompanied by the upregulation of the NOX complex subunit, p47^phox^. This preliminary study demonstrates that PARPi‐induced oxidative stress correlates with Ets‐1 expression, which might represent a biomarker potentially able to identify the most sensitive cancer cells for treatment with PARPi.

## Author Contributions

**Magalie Hervieu:** investigation, data curation, formal analysis, writing – original draft. **Arnaud J. Legrand:** conceptualization. **Emilie Floquet:** investigation, data curation. **Thierry Idziorek:** data curation. **Corentin Spriet:** data curation. **Didier Monté:** data curation. **Vincent Villeret:** visualization, writing – review and editing. **Marc Aumercier:** conceptualization, methodology, formal analysis, data curation, visualization, writing – original draft, writing – review and editing, funding acquisition, supervision. **Souhaila Choul‐li:** methodology, validation, formal analysis, data curation, visualization, writing – original draft, writing – review and editing, supervision.

## Conflicts of Interest

The authors declare no conflicts of interest.

## Data Availability

The data that support the findings of this study are available from the corresponding author upon reasonable request.
